# Factors predicting recurrence after curative resection for rectal cancer: a 16-year study

**DOI:** 10.1186/s12957-019-1718-1

**Published:** 2019-10-28

**Authors:** Waad Farhat, Mohamed Azzaza, Abdelkader Mizouni, Houssem Ammar, Mahdi ben Ltaifa, Sami Lagha, Mohamed Kahloul, Rahul Gupta, Mohamed Ben Mabrouk, Ali Ben Ali

**Affiliations:** 1 Department of Gastrointestinal Surgery, Sahloul Hospital, Sousse, Tunisia; 2 Department of Anesthesia and Intensive Care, Sahloul Hospital, Sousse, Tunisia; 3 Department of Gastrointestinal Surgery, Synergy Institute of Medical Sciences, Dehradun, India

**Keywords:** Rectal adenocarcinoma, Recurrence, Prognosis

## Abstract

**Background:**

The recurrence after curative surgery of the rectal adenocarcinoma is a serious complication, considered as a failure of the therapeutic strategy. The aim of this study was to identify the different prognostic factors affecting the recurrence of adenocarcinoma of the rectum.

**Methods:**

A retrospective analysis of patients operated for adenocarcinoma of the rectum between January 2000 and December 2015 was conducted. The study of the recurrence rate and prognostic factors was performed through the Kaplan Meier survival curve and the Cox regression analysis.

**Results:**

During the study period, 188 patients underwent curative surgery for rectal adenocarcinoma, among which 53 had a recurrence. The recurrence rate was 44.6% at 5 years. The multivariate analysis identified four parameters independently associated with the risk of recurrence after curative surgery: a distal margin ≤ 2 cm (HR = 6.8, 95% CI 2.7–16.6, 6), extracapsular invasion of lymph node metastasis (HR = 4.4, 95% CI 1.3–14), tumor stenosis (HR = 4.3, 95% CI 1.2–15.2), and parietal invasion (pT3/T4 disease) (HR = 3, 95% CI 1.1–9.4).

**Conclusion:**

The determination of the prognostic factors affecting the recurrence of rectal adenocarcinoma after curative surgery allows us to define the high-risk patients for recurrence.

**Trial registration:**

ClinicalTrials.gov Identifier: NCT03899870. Registered on 2 February 2019, retrospectively registered.

## Introduction

Colorectal cancer is one of the most frequently diagnosed cancers and a major cause of cancer deaths worldwide [1]. Recurrence after curative surgery is one of the major factors affecting the long-term survival and its frequency is estimated to be 22.5% at 5 years, of which 12% have a local recurrence. The overall survival in case of recurrence is about 11% at 5 years [2]. Several patient-, tumor-, and treatment-related prognostic factors are associated with the risk of recurrence of rectal adenocarcinoma. Some of these factors such as TNM stage [3], lymphatic and perineural invasion [3, 4], and vascular emboli [5, 6] have been found to affect recurrence-free survival in most studies. While the impact of other factors such as distal resection margin [7], tumor size [8, 9], extracapsular spread [10], and neoadjuvant chemoradiotherapy [11, 12] on recurrence remains controversial. Most of the previous studies on prognostic factors have been from American and European countries with very little data from African countries. Recognition of these factors helps in the identification of high-risk patients who require close and more rigorous postoperative surveillance. Hence, this study was conducted to determine the factors affecting recurrence after curative resection of rectal cancer in the African population.

## Patients and methods

This is a retrospective study of prospectively maintained data of all the patients who underwent curative resection for rectal adenocarcinoma over 16 years (between January 2000 and December 2015) at the Department of Digestive and Visceral Surgery of Sahloul Hospital, Sousse, Tunisia. We excluded patients who underwent palliative surgery, patients with microscopically or macroscopically positive resection margin (proximal, distal, or circumferential), patients with tumors other than adenocarcinoma, and those who died in the postoperative period because of complications. Informed consent was taken from all patients. This study was approved by the research ethics board of Sahloul Hospital and has been performed in accordance with the ethical standards laid down in the 1964 Declaration of Helsinki and its later amendments.

### Preoperative staging

1. T staging— On MRI pelvis, tumor extending into the perirectal fat was labeled as T3 disease and those invading adjacent organs were considered to have T4 disease [13].
2. Nodal staging—On MRI pelvis, heterogeneity of signal intensity on T2W sequences or irregular margins of the lymph nodes or lymph node size > 8 mm was considered to be pathological [13].

### Neoadjuvant therapy

Patients with locally advanced disease (cT3, cT4) or lymph nodal positive disease were offered neoadjuvant therapy. In the neoadjuvant therapy, we used 45 Gy in 25 fractions with concurrent 5-fluorouracil [5-FU] infusion (600 mg/m^2^) [14]. Patients were operated 8 to 10 weeks after neoadjuvant therapy [15, 16]. Sometimes, for the elderly patients with multiple co-morbidities, we used short-course pelvic radiation therapy which included 25 Gy in five fractions over 1 week.

### Surgery

Patients with tumors in the upper and middle third rectum underwent anterior and low anterior resection, respectively. Patients with tumors in the lower third of the rectum where anal sphincters could not be preserved underwent abdominoperineal resection. In most of the cases, inferior mesenteric artery (IMA) was ligated caudal to the origin of the left colic artery to preserve the autonomic nerves at the IMA origin and maintain a good blood supply to the left colon and the anastomotic site. For the tumors of the upper rectum, partial excision of the mesorectum was performed up to a minimum of 5 cm from the inferior aspect of the tumor. For the tumors of the middle and low rectum, a total mesorectum excision was done with the minimum distal mucosal margin of 1 to 2 cm. In cases where coloanal anastomosis was performed, an ileostomy was made. However, after low colorectal anastomosis, an ileostomy was performed if the colon was poorly prepared or the anastomotic leak test was positive.

In most of the cases, open surgery was performed. Laparoscopic surgery was performed in selected cases. Wide local excision was performed in selected cases with T1 tumors without locoregional lymphadenopathy.

### Adjuvant therapy

Patients with locally advanced disease (pT3, T4) or lymph nodal positive disease were offered adjuvant therapy. In most of the cases, FOLFOX (leucovorin, 5**-**FU, oxaliplatin) regimen was used and for elderly patients who could not tolerate this regimen, we used oral capecitabine.

### Follow-up

Postoperative follow-up included clinical examination, serum carcinoembryonic antigen (CEA) levels, and abdominal ultrasound every 3 months during the first 2 years and then every 6 months for 2 years. We also performed a thoraco-abdominopelvic CT scan every 6 months for the first 2 years and then every year for 3 years. Colonoscopy was done after 1 year, and if it was normal, it was repeated after 3 years.

### Definitions

Adenocarcinoma of the rectum was defined as cancer arising from the glandular cells of the rectal epithelium and located anatomically within the last 15 cm of the digestive tract from the anal canal.

Recurrence was defined as the development of any new malignant lesion within the field of surgery (locoregional recurrence) or outside it (distant metastasis) after initial resection was judged to be curative (R0) based on the preoperative imaging and histopathological examination of the resected specimen. The confirmation of the recurrence was made by pathological examination of a biopsy performed during an endoscopic examination, a laparotomy, computed tomography-guided biopsy, or finally at autopsy.

### Statistical analysis

The qualitative variables were summarized by simple and relative frequencies. Quantitative variables were expressed as the median and interquartile interval. The frequency of recurrence of rectal adenocarcinoma was calculated by the incidence density and using the Kaplan-Meier survival analysis. All the variables associated with recurrence with *p* < 0.20 on univariate analysis were integrated into a Cox regression model that allowed the identification of prognostic factors independently associated with recurrence by calculating the hazard ratio (HR) with their 95% confidence intervals. The mean and median time to recurrence was calculated using the Kaplan-Meier curve. The log-rank test was used to compare the Kaplan-Meier curves for the prognostic factors independently associated with rectal adenocarcinoma recurrence.

## Results

During the study period, 269 patients were operated for rectal adenocarcinoma. Out of these, 188 patients were included in this study. Fifty-three patients developed recurrence in the follow-up period. The basic characteristics of the study population were summarized in Table 1. The sex ratio was 1.35 and the mean age was 60.5 years (50–71.7) for the total population versus a sex ratio of 0.8 and a median age of 60 years (45.5–70) for patients who had a recurrence.

**Table 1:** Patient and tumor characteristics

Characteristic	Number of patients [*n* (%)]
Number of patients in this study	188
Age (years) (mean)	60.5
Sex ratio (M:F)	1.35:1
Location of tumor	
Upper rectum	45 (24)
Middle rectum	65 (35)
Lower rectum	78 (41)
cT stage	
T1/T2	24 (13)
T3	155 (82)
T4	9 (5)
cN stage	
N0	82 (44)
N+	106 (56)
cTNM stage	
I	49 (27)
II	38 (20)
III	98 (51)
IV	3 (2)
Parietal invasion	
pT1–T2	64 (34)
pT3–T4	124 (66)
pT stage	
T1	14
T2	50
T3	107
T4	17
pN stage	
N0	127
N1	34
N2	17
N3	10
pTNM stage	
I	56 (30)
II	68 (37)
III	61 (32)
IV	3 (1)
Tumor perforation	7 (4)
Invasion of surrounding organs	16 (8)
Degree of differentiation	
Well	41 (22)
Moderate	133 (71)
Poor	13 (7)
Neoadjuvant therapy	120 (64)
Type of surgery	
APR	35 (19)
Low anterior resection	144 (76)
Local excision	9 (5)
Laparoscopic surgery	25 (13)
Postoperative complications	39 (21)
Adjuvant therapy	65 (34)
Number of patients with recurrence	53 (28)
Median follow-up period	57 months
Median recurrence-free survival	54 months
Median survival after recurrence	25 months
Median overall survival	75 months
2-year overall survival	73%
5-year overall survival	54%

**Fig. 1:**
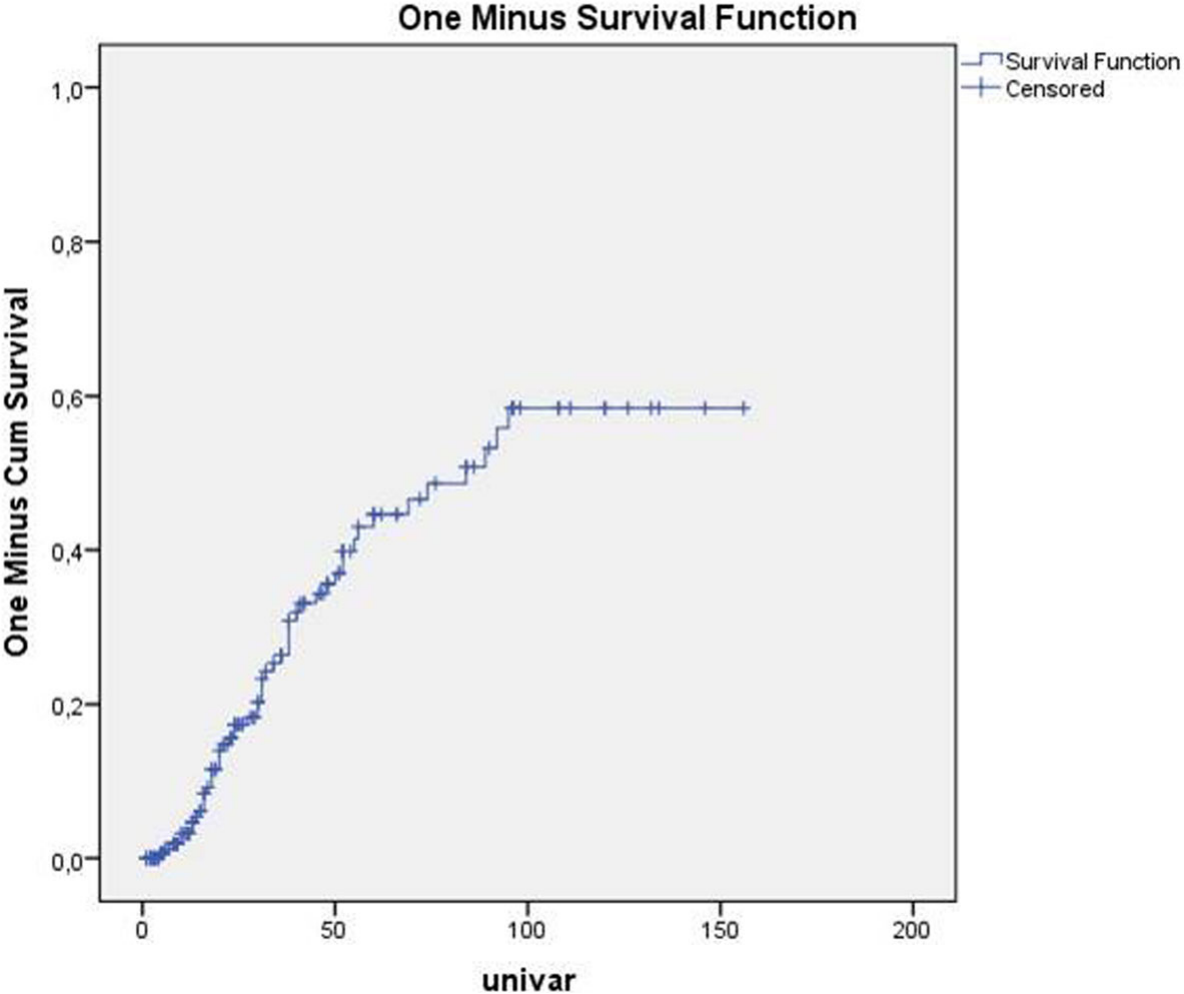
predicting recurrence rate in function of time after curative resection for rectal adenocarcinoma

The median recurrence-free survival was 54 months. The recurrence rate was 44.6% at 5 years and 58.4% at 10 years (Fig. 1). The incidence density of recurrence was 1/10 patient-year.

The recurrence rate was 17% (9 cases) for tumors of the upper rectum versus 30% (16 cases) for tumors of the middle rectum and 50.9% (27 cases) for tumors of the lower rectum (Table 2). The recurrence rate was 24.5% (13 cases) after abdominoperineal resection versus 67.9% (36 cases) after anterior resection of the rectum and 7.5% (4 cases) after local excision.

**Table 2:** Demographic and clinical characteristics of the patients with and without recurrence

	Without recurrence, *n* = 135		Recurrence, *n* = 53		Total, *N* = 188	
	*N*	%	*N*	%	*N*	%
Demographic characteristics						
Sex						
Male	84	62.2	24	45.3	108	57.4
Female	51	37.8	29	54.7	80	42.6
Age						
< 50 years	23	17.0	18	34.0	41	21.8
≤ 50 years	112	83.0	35	66.0	147	78.2
Clinical characteristics						
Location of the tumor						
Upper rectum	36	26.7	9	17.0	45	23.9
Middle rectum	49	36.3	16	30.2	65	34.6
Low rectum	50	37.0	28	50.9	77	41.0
Size of the tumor						
< 5 cm	92	68.1	24	45.2	116	61.7
> 5 cm	43	31.1	29	54.8	72	38,3
Stenotic character						
Yes	21	15	13	24.6	34	18
No	114	85	40	75.4	154	82
CEA levels						
Normal	26	19.2	24	45.2	50	27
Abnormal	109	80.8	29	54.8	138	73
Neoadjuvant therapy						
Yes	85	62.9	35	66	120	63.8
No	50	37.1	18	34	68	36.2
Surgical intervention						
Abdominoperineal resection	22	15.6	13	24.5	35	18.1
Anterior resection of the rectum	108	70.4	36	67.9	144	77.1
Local excision	5	4.4	4	7.5	9	4.8
Distal margin						
≤ 2 cm	37	27.4	31	58.4	68	37
> 2 cm	90	72.6	22	41.6	112	63
Intraoperative incident						
Yes	32	23.7	17	32	49	26
No	103	76.3	36	68	139	64
pT stage						
T1–T2	54	40	10	18.8	64	34
T3–T4	81	60	43	81.2	124	66
Invasion of neighboring organs						
Yes	6	4	10	18.8	16	8.5
No	129	96	43	81.2	172	11.5
Tumor perforation						
Yes	2	1.5	5	9.4	7	3.7
No	133	98.5	48	90.6	181	96.3
pN stage						
N0	106	78.5	21	39.6	127	67.5
N+	29	21.5	32	60.4	61	32.5
Extra capsular invasion						
Yes	8	6	31	58.4	39	20.7
No	127	64	22	41.6	149	79.3
Vascular emboli						
Yes	10	7.4	20	37.7	30	16
No	125	92.6	33	62.3	158	84
Lymphatic and perineural invasion						
Yes	7	5.2	14	26.4	21	11.1
No	128	94.8	39	73.6	167	88.9
pTNM stage						
I and II	102	75.5	22	41.5	124	66
III and IV	33	84.5	31	58.5	64	34

Table 3 describes the characteristics of recurrence. The most common symptom of recurrence was pelvic pain (73.6%). The diagnosis of recurrence was made by abdominal CT scan in 47.1% of cases, with pelvic MRI in 13.2% and with endoscopy in 39.6% of cases. Locoregional recurrences were mainly located in the pelvis in 35.8%, at the anastomotic site in 18.7%, and both places in 13.2% cases. Distant metastases were located in the liver in 15.1% cases and the lung in 17.2% cases (Table 3).

**Table 3:** Characteristics of the recurrent disease

	*n*	%
Presenting symptoms/signs		
Pelvic pain	39	73.6
Alteration of the general condition	31	58.5
Mass at digital rectal examination	20	37.7
Means of diagnosis of recurrence		
Abdominal and pelvic CT scan	25	47.1
Digestive endoscopy	21	39,6
Pelvic MRI	7	13.2
Recurrence location		
Locoregional recurrence		
Pelvic	25	47.1
Anastomotic	8	15.0
Pelvic and anastomotic	10	18.8
Remote metastasis		
Hepatic	15	15.1
Pulmonary	9	17.0
Bone	3	5.6
Brain	1	1.8
Ovarian	1	1.8

Table 4 summarizes the univariate analysis of all the prognostic factors likely to affect the development of recurrence. The multivariate analysis (Table 5) revealed four independent parameters associated with the risk of recurrence after curative surgery: a distal margin ≤ 2 cm (HR = 6.8, 95% CI 2.7–16.6), extracapsular spread (HR = 4.4, 95% CI 1.3–14), tumor stenosis (HR = 4.3, 95% CI 1.2–15.2), parietal invasion according to the TNM classification (HR = 3, 95% CI 1–9.4).

**Table 4:** Univariate analysis to determine the factors associated with recurrence

Prognostic factors	Risk category	Reference category	Univariate analysis		
			HR	95% CI	*p*
Factors related to the population					
Sex	Female	Male	1.651	0.9–2.8	0.07
Age	< 50 years	≥ 50 years	1.136	0.6–2.0	0.66
Factors related to the tumor					
Location	Low rectum	Upper or middle	1.129	0.7–2.2	0.35
Fixity	Mobile	Fixed	1.084	0.5–2.0	0.80
Stenotic character	Stenotic	Non-stenotic	*2.203*	*1.1–4.2*	*0.01*
Size	≥ 5 cm	< 5 cm	1.496	0.8–2.6	0.15
Biologic factors					
CEA levels	Abnormal	Normal	0.721	0.3–1.3	0.32
LDH levels > 400	Present	Absent	0.673	0.1–3.7	0.65
Factors related to adjuvant and neoadjuvant treatment					
Neoadjuvant radiotherapy	Absent	Present	1.337	0.7–2.3	0.31
Neoadjuvant radiochemotherapy	Absent	Present	1.551	0.8–2.9	0.17
Adjuvant chemotherapy	Absent	Present	1.308	0.7–2.3	0.37
Factors related to surgery					
Urgency of the intervention	Yes	No	7.027	0.9–52.6	0.05
Experience of the surgeon	Junior	Senior	0.888	0.3–1.9	0.77
Surgical approach	Laparoscopic	Laparotomy	0.962	0.3–2.7	0.94
Surgical intervention	Abdominoperineal resection	Anterior resection of the rectum	0.648	0.3–1.2	0.18
Vascular ligation	1 cm from the origin	At the origin	1.334	0.7–2.3	0.33
Distal margin	≤ 2 cm	> 2 cm	*2.792*	*1.5–4.9*	*0.00*
Intraoperative incident	Yes	No	*3.716*	*1.6–8.4*	*0.00*
Anastomotic fistula	Present	Absent	0.637	0.2–1.4	0.63
Pathological factors					
Parietal invasion (pT stage)	T3–T4	T1–T2	*2.278*	*1.1–4.5*	*0.02*
Invasion of neighboring organs	Yes	No	*3.040*	*1.5–6.0*	*0.00*
Tumor perforation	Yes	No	*4.128*	*1.6–10.5*	*0.00*
Ganglionic invasion (pN stage)	Yes	No	*2.677*	*1.5–4.6*	*0.00*
Extra capsular invasion	Yes	No	*2.789*	*1.1–6.6*	*0.02*
Vascular emboli	Yes	No	*3.817*	*2.1–6.6*	*0.00*
lymphatic and perineural invasion	Yes	No	*4.235*	*2.2–7.8*	*0.00*
Mucoid colloid component	Yes	No	1.360	0.7–2.6	0.34
pTNM stage	III and IV	I and II	*2.833*	*1.6–4.8*	*0.00*

*HR* hazard ratio, *CI* confidence interval

**Table 5:** Multivariate analysis to identify the prognostic factors for recurrence in rectal adenocarcinoma

Prognostic factors	Risk category	Reference category	Multivariate analysis		
			HR	95% CI	*p*
Factors related to the population					
Sex	Female	Male	1.356	0.5–3.3	0.50
Age	< 50 years	≥ 50 years	0.429	0.1–1.0	0.07
Factors related to the tumor					
Stenotic character	Stenotic	Non-stenotic	*4.387*	*1.2–15.2*	*0.02*
Size	≥ 5 cm	< 5 cm	0.710	0.2–1.7	0.45
Factors related to adjuvant and neoadjuvant treatment					
Neoadjuvant radiochemotherapy	Absent	Present	1.502	0.6–3.3	0.32
Factors related to surgery					
Surgical intervention	Abdominoperineal resection	Anterior resection of the rectum	1.216	0.4–3.1	0.69
Distal margin	≤ 2 cm	> 2 cm	*6.819*	*2.7–16.6*	*0.00*
Intraoperative incident	Yes	No	1.627	0.2–9.0	0.57
Pathological factors					
Parietal invasion (pT stage)	T3–T4	T1–T2	*3.073*	*1.1–9.4*	*0.05*
Perforated character of the tumor	Yes	No	2.279	0.1–33.6	0.54
Ganglionic invasion (pN stage)	Yes	No	0.300	0–6.7	0.44
Extra capsular invasion	Yes	No	*4.408*	*1.3–14.0*	*0.01*
Vascular emboli	Yes	No	1.398	0.5–3.4	0.47
lymphatic and perineural invasion	Yes	No	2.369	0.7–7.0	0.12
pTNM stage	III and IV	I and II	4.623	0.2–90.9	0.31

*HR* hazard ratio, *CI* confidence interval

**Fig. 2:**
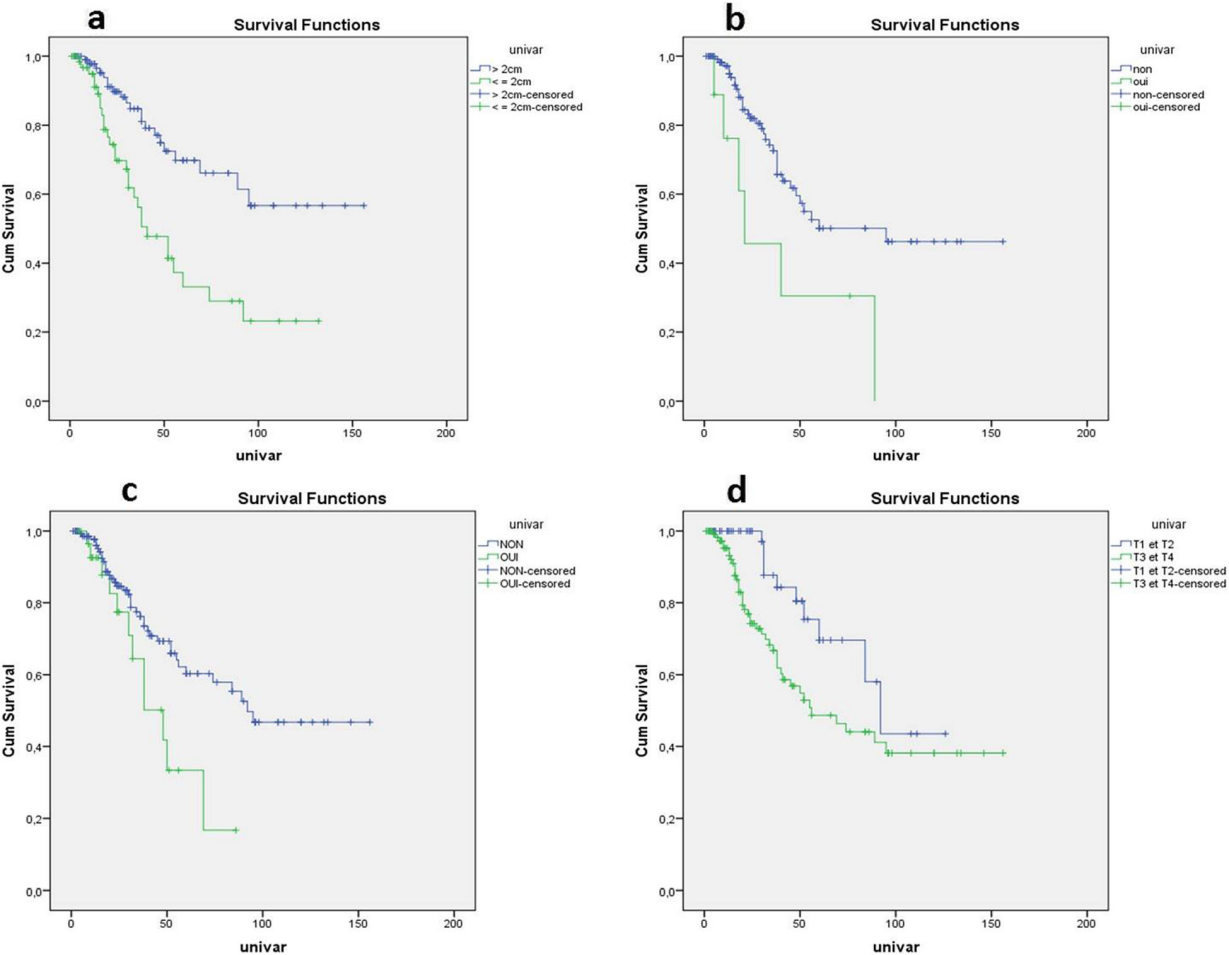
Kaplan-Meier curves comparing the recurrence-free survival of the patients according to the four parameters independently associated with the risk of recurrence after curative surgery. **a** Distal margin (log-rank = < 10^− 3^). **b** Extracapsular invasion of lymph node metastasis (log-rank = 0.015). **c** Tumor stenosis (log-rank = 0.016). **d** Parietal invasion according to the TNM classification (log-rank = 0.016)

Figure 2 illustrates the effect of these four prognostic factors on the recurrence-free survival rate. The mean recurrence-free survival was 59.7 ± 7.53 months for patients with the distal margin ≤ 2 cm versus 109.7 ± 8.28 months for those patients with the distal margin > 2 cm. The presence of extracapsular spread reduced the mean recurrence-free survival from 95 months to 21 months. The non-stenotic nature of the tumor increased the mean recurrence-free survival from 48 months to 92 months. In those with parietal invasion, the mean recurrence-free survival was 92 months for T1 and T2 tumors whereas it was only 56 months for T3 and T4 tumors.

## Discussion

Recurrence after curative surgery of the rectal adenocarcinoma is a serious health problem not only because of its high frequency but also because it considerably impacts of the life expectancy and the quality of life of the patients [1]. Several prognostic factors related to the patient, tumor, and therapeutic protocol (radio preoperative chemotherapy, surgical procedure, and adjuvant chemotherapy) affect the development of recurrence. Determination of these prognostic factors can help define high-risk patients who require more frequent postoperative surveillance and adjuvant therapy.

According to our study, the recurrence rate was estimated to be 44.6% at 5 years. The reported recurrence rate at 5 years is 25–37% which is slightly lower than that of this study [17, 18]. A meta-analysis by Puhlman [19] reported the 5-year recurrence rate between 23% and 41% with a mean of 27%.

Our results reflect the seriousness of the problem of recurrence after curative surgery of rectal adenocarcinoma in central Tunisia. Several factors could explain the increased risk of recurrence, particularly the disintegration of oncological and radiotherapy care services.

On multivariate analysis according to the Cox regression model, four prognostic factors for recurrence of rectal adenocarcinoma after curative surgery were identified: distal resection margin ≤ 2 cm (HR = 6.8, 95% CI 2.7–16.6), extracapsular invasion (HR = 4.4, 95% CI 1.3–14), tumor stenosis (HR = 4.3, 95% CI 1.2–15.2), and degree of parietal invasion (T3/T4) (HR = 3, 95% CI 1.1–9.4).

As with all the cancers, the level of resection must be at a maximum distance from the tumor to achieve at least 2 cm of macroscopic tumor-free resection margin during the surgical procedure. The macroscopic limit of a tumor lesion is often exceeded at the microscopic level by submucosal tumor invasion. If the resection limit is microscopically invaded by the tumor, it will affect the long-term outcome as it will be a potential site for local recurrence. For tumors of the lower and middle rectum, studies have shown that there is no additional reduction in the risk of local recurrence with a distal margin greater than 2 cm below the lower end of the tumor [7–11].

Currently, a 1-cm distal margin has been suggested by several randomized studies, especially after neoadjuvant therapy [11, 12] to the safe limit [20]. Some studies [21, 22] compared oncology results between a distal resection margin ≤ 1 cm and > 1 cm and did not show a significant difference in terms of the recurrence rate. In our study, a distal resection margin ≤ 2 cm was an independent predictor of recurrence on multivariate analysis.

The second important factor found in our study was extracapsular invasion which increased the risk of recurrence by fourfold. This would be explained by the fact that the lymph node, which is the first defense barrier of the body to prevent the lymphatic spread, does not control tumor invasion. Thus, the residual tumor cells lying outside the lymph nodes may become the site of tumor recurrence. Few studies have investigated extracapsular invasion in the analysis of risk factors for tumor recurrence. It has been studied in the cancers of other sites such as the vulva, lungs, and some digestive cancers such as the esophagus and stomach. In most of these studies, the extracapsular invasion was found to be a bad prognostic factor [10]. In colorectal cancer, because of the conflicting results in the reported studies, this parameter does not appear in TNM classification, nor standardized pathological reports [23]. A retrospective study carried out in Tunisia including 75 cases of colorectal adenocarcinoma concluded that the extracapsular invasion of lymph node metastasis correlated with the occurrence of local recurrences (*p* = 0.001) and metachronous metastases (*p* = 0.01). Our series confirms the prognostic value of extracapsular invasion in rectal adenocarcinoma.

The stenosing character of the tumor was identified in our study as a risk factor for the development of recurrence in rectal cancer. The tumor stenosis indicates that the disease process is going on for quite some time that exposes the patient to the risk of tumor dissemination and may explain the increased risk for locoregional and distant recurrence. In a study by Larsen et al. [24] including 254 patients with T3 and T4 rectum cancers, the stenosing character of the tumor was associated with a higher rate of recurrence (*p* = 0.005) and a lower survival rate (*p* = 0.005) (0.01). In another study by Chapet et al. [25], the stenosing character of the tumor was not an independent prognostic factor for recurrence, but the 5-year survival rate was reduced to 12%. In our series, the stenosing character of the tumor was significantly associated with the risk of recurrence, thus concordant with the results of Larsen et al. [24].

Another important prognostic factor for recurrence found in this study was the degree of parietal infiltration. The vascular channels are present in the third layer of the rectal wall which corresponds to T3 of parietal infiltration according to the TNM classification. Any invasion of the rectal wall beyond this stage is associated with vascular invasion and therefore with a higher risk of dissemination and recurrence. The degree of parietal invasion was one of the five known prognostic factors for rectal cancer which were identified at a North American consensus conference [26].

Besides, several studies have shown that the degree of parietal invasion was significantly associated with the risk of recurrence [27, 28]. A recent study by Chen et al. involving 359 patients operated for rectal cancer showed that the T3 stage has a higher risk of recurrence compared to the T2 stage (*p* = 0.012) [3]. Our study confirms that the degree of parietal invasion is an independent predictor of recurrence.

In the literature, other prognostic factors for recurrence have been noted but not found significant in this study. These factors are related to the quality of the surgical procedure [29–31] such as total mesorectal excision, neoadjuvant treatment [32, 33], preoperative radiochemotherapy, and histopathological criteria of the tumor such as the number of lymph nodes involved, the perineural invasion, and the vascular emboli [34].

The knowledge of the prognostic factors for recurrence of rectal adenocarcinoma after curative surgery could calculate a predictive risk score. This score will define a profile of patients at very high risk of recurrence who need a surveillance protocol that differs from the recommended guidelines.

This study is limited by its retrospective nature. However, the medical records specific to the tumor pathologies were very well preserved (operating records, radiological, pathological, and other details); thus, the risk of information bias attributed to the loss of records is less and does not affect the reliability of the results.

## Conclusion

This study revealed distal resection margin ≤ 2 cm, extracapsular invasion of lymph node metastasis, tumor stenosis, and degree of parietal invasion ≥ T3 to be independent prognostic factors for recurrence of rectal adenocarcinoma in African patients. Future multicentric national scale studies are required to validate the results of this study.

This study was approved by the Sahloul Hospital ethical committee. All procedures performed in studies involving human participants were in accordance with the ethical standards of the institutional and/or national research committee (include name of committee + reference number) and with the 1964 Helsinki declaration and its later amendments or comparable ethical standards.

## Data Availability

Access to the data and the calculation method can be obtained from the authors by email (hosshoss24@hotmail.fr).
